# P-799. Potential Impact of Flexible Respiratory Testing: Advancing Diagnostic Stewardship Through Targeted Diagnostics

**DOI:** 10.1093/ofid/ofaf695.1009

**Published:** 2026-01-11

**Authors:** Bridget Parsons, James Snyder

**Affiliations:** Diasorin, Austin, Texas; University of Louisville Medical Center, KY

## Abstract

**Background:**

Respiratory pathogens present a significant public health challenge contributing to healthcare costs and patient morbidity. Traditional syndromic panel testing often leads to broad testing for rare pathogens and inefficiencies in laboratory utilization. Flexible testing allows clinicians to target pathogens based on real-time epidemiology, supporting diagnostic stewardship efforts. Additionally, laboratory efficiency can be improved by reducing unnecessary testing. This analysis examines the epidemiology of respiratory pathogens in the University of Louisville patient population and the potential economic and operational impact of a flexible, diagnostic stewardship-aligned testing approach.

**Methods:**

A retrospective analysis was conducted on respiratory syndromic test results from adult patients at The University of Louisville between January and December 2024. A total of 4,346 specimens were included. The frequency of detected pathogens was summarized, and a flexible testing model using the LIAISON PLEX® Respiratory Flex Assay (Diasorin) was applied retrospectively by prioritizing the most prevalent targets. Cost implications and laboratory workflow impact were assessed.

**Results:**

A total of 4,346 respiratory tests were conducted in 2024, with an average positivity rate of 18% (N=799). The seven most common pathogens accounted for 94% of all positive results. Human metapneumovirus emerged as a notable seasonal pathogen, comprising 6% of positive detections in the spring and summer. A flexible testing model prioritizing the top seven pathogens could have reduced full syndromic panel utilization by 94%, with only 6% of cases requiring expanded testing. Workflow impact assessment is ongoing and will be presented.

**Conclusion:**

This analysis demonstrated that 94% of positive cases in 2024 were attributable to seven pathogens, indicating that a limited, flexible panel could address most clinical needs. Applying this approach would have reduced full panel use by 94%, supporting more efficient resource utilization. These findings suggest that flexible respiratory testing may offer a scalable strategy to improve diagnostic stewardship and laboratory efficiency, particularly in high-volume or resource-limited settings.

**Disclosures:**

All Authors: No reported disclosures

